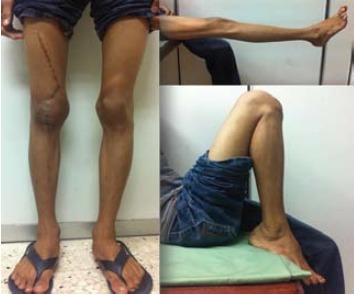# Reimplantation of an Extruded Femoral Segment After Gamma Sterilization in A Type IIIA Supracondylar Femur Fracture: A Case Report

**DOI:** 10.5704/MOJ.1407.016

**Published:** 2014-07

**Authors:** N Aizah, Y Su, CWM Shaifulnizam, MA MRos

**Affiliations:** Department of Orthopaedics, Hospital Kemaman, Terengganu, Malaysia; Department of Orthopaedics, Hospital Kemaman, Terengganu, Malaysia; Department of Orthopaedics, Hospital Kemaman, Terengganu, Malaysia; Department of Orthopaedics, Hospital Kemaman, Terengganu, Malaysia

## Abstract

Extruded bone is a rare complication of high energy open fractures, and there is only a handful of literature on reimplantation of the extruded segment. No clear guidelines exist regarding timing of reimplantation, stabilization of extruded bone segments, and also bone disinfection and sterilization techniques. Previous reports describe sterilization using thermal or chemical methods. We present a case of successful reimplantation of an extruded metaphyseal segment of femur after gamma sterilization in a fourteen- year old boy.

**Key Words:**

extruded bone, reimplantation, gamma sterilization

## Introduction

The optimal management of severe open fractures remains a problem due to high rates of infection and delayed union. These risks are further amplified when high-energy open fractures result in significant bone loss. Large defects may necessitate the use of large volume allografts, vascularized bone grafts or distraction osteogenesis for reconstruction.

In a scenario where a fragment of extruded bone is available for reimplantation, the many benefits include maintenance of skeletal and soft tissue length, averting the morbidity associated with autograft harvest and obviating the need for allograft bone or prolonged bone transport procedures. However, there is a heightened risk of infection with the reimplantation of an extruded, contaminated and devascularized bone segment.

To reduce the risk of infection, several sterilization techniques of an extruded bone segment have been reported including thermal and chemical sterilization. Here we describe successful reimplantation of an extruded femoral segment after sterilization with gamma irradiation in an adolescent. We also review the various methods of sterilization of extruded bone segments used in the management of open fractures.

## Case Report

A 14-year old, otherwise healthy boy presented following a road crash in which he was a motorcyclist hit by a car. On presentation, he was alert, with major injuries restricted to his right lower limb. He had sustained type IIIA open supracondylar fracture of the right femur (OTA 33-A3.1) with bone loss. There was a 5x3cm longitudinal laceration wound over the medial aspect of the distal right thigh. Otherwise perfusion to the limb was good and distal pulses were present. Brought in along with the patient was an extruded split segment of the metaphyseal femur measuring 8x2x3cm (Figure 1).

Initial treatment consisted of massive irrigation of the wound with saline solution to remove visible debris, and commencement of intravenous antibiotic therapy with cefuroxime, gentamicin, and metronidazole per protocol. The retrieved bone segment was washed with copious amount of normal saline and visible contamination was curetted before storage at 4oC prior to irradiation.

Due to the limitations faced by a small hospital, initial debridement in the operating theatre was only accomplished 22 hours after the initial injury. The index procedure included aggressive debridement and irrigation of the open fracture wound followed by fixation of the remaining segment of the metaphysis to the femoral diaphysis using two lag screws.

Surgical wound extensions were closed primarily while the traumatic wound was left open. A proximal tibial pin was inserted and the patient was put on Bohler-Braun traction in ward.

Simultaneously, the extruded bone segment was irradiated with a dose of 25kGy at the Malaysian Nuclear Agency.

Two weeks after the initial accident, the bone segment was reimplanted and stabilized with a distal femur locking plate (Synthes USA, West Chester, PA), with delayed primary closure of the traumatic wound. The immediate postoperative period was uneventful apart from post-operative fever which settled with intravenous antibiotics. The patient was discharged well a week after his second operation.

**Fig. 2: Radiographs at 1 year showing incorporation of the graft with fracture union:**
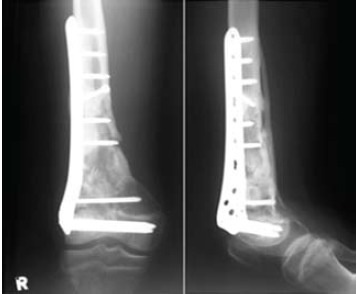


Ensuing outpatient follow-up showed excellent wound healing. At three months post-operative, there was radiographic evidence of bridging fracture site callus. Partial weight bearing was then allowed. At six months post operatively, radiographic evidence of fracture union was achieved and complete weight bearing on the affected limb was allowed (Figure 2). With physiotherapy, the patient also regained full knee range of knee motion 0’ – 150’, comparable to the opposite side (Figure 3). At one-year follow-up, he could resume all pre-injury activities, and was pain-free.

## Discussion

Many factors contributed to the successful bone reimplantation in this patient, including the distal femoral location of the bone loss, meticulous wound debridement and care, sufficient antibiotic coverage, adequate sterilization of the extruded fragment, delayed reimplantation and definitive fixation as well as the patient’s young age and otherwise excellent health.

A literature search turned up only a handful of similar cases. No clear guidelines exist on sterilization methods for traumatically extruded bone segments intended for reimplantation.

The earliest report of such a case was in 1965 when Kirkup described successful replacement of a nine-inch meta-diaphyseal femoral segment after boiling and autoclaving ^1^. Although complicated with chronic osteomyelitis with the presence of an involucrum radiographically, subsequent complete reincorporation of the autogenous “bone graft” was noted.

More recently, Rouvillain ^2^ and Marzurek ^3^ reported similar success in reimplantation of traumatically extruded metadiaphyseal femoral segments. Rouvillain sterilized an extruded 11cm meta-diaphyseal femur by autoclaving the bone segment at 121oC, 1.3 bars for 20 minutes, while Marzurek reported chemical sterilization of a 13cm meta-diaphyseal femur with chlorhexidine 4% soak for a total of 270 minutes. Timely fracture union with full functional recovery was also achieved in both cases.

Thermal sterilization by autoclave is a readily available and well established method, but destroys bone osteo-inductivity and largely decreases its mechanical strength ^4^. On the other hand, various studies on chemical sterilization methods show conflicting results regarding sterilization efficacy ^5^ and there are no clear guidelines regarding substance concentration and duration for exposure. Being unfamiliar with the efficacy of both these methods, we opted for sterilization via gamma irradiation, as this is the gold standard in bone banking for sterilization of allograft bone. The Malaysian Nuclear Agency received and sterilized the bone segment with an irradiation dose of 25kGy in accordance to ISO 11137, 2002.

When faced with extrusion of a large segment of bone, the management of each case should be individualized with consideration given to reimplantation of the extruded segment. In weighing methods of sterilization, the prevention of infection, in most cases, is a more important determinant of patient outcome than implanted bone segment viability. Where feasible, gamma irradiation can be considered for sterilization of the contaminated extruded bone.

**Fig. 1: Injury (A) anteroposterior and (B) lateral radiographs demonstrating a distal femoral defect and (C) the extruded bone segment after being washed with saline:**
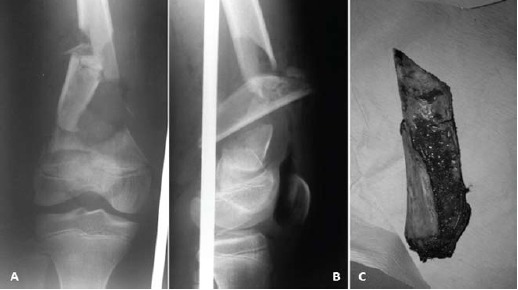


**Fig. 3: Photograph of the leg at 1 year follow-up with full active ROM:**